# *Tribulus terrestris* L.: a medicinal plant with promising therapeutic potential for skin diseases

**DOI:** 10.3389/fphar.2025.1732016

**Published:** 2026-01-12

**Authors:** Xiao-Mu Wang, Xiao-Min Liu, Yan Zeng, Ke-Jing Zhu, Tian-Tian Shen, Fang Bian

**Affiliations:** 1 Drug Clinical Trial Institution, Xiangyang Key Laboratory of Special Preparation of Vitiligo, Xiangyang Central Hospital, Affiliated Hospital of Hubei University of Arts and Science, Xiangyang, Hubei, China; 2 Department of Pharmacy, Xiangyang Key Laboratory of Special Preparation of Vitiligo, Xiangyang Central Hospital, Affiliated Hospital of Hubei University of Arts and Science, Xiangyang, Hubei, China

**Keywords:** advantages and limitations, Chinese medicine, skin diseases, therapeutic potential, *Tribulus terrestris* L

## Abstract

Tribulus *terrestris* L. (*T. terrestris* L.), a traditional medicinal plant, has garnered increasing attention for its potential in treating skin disease. This review comprehensively summarizes current research on the protective effects of *T. terrestris* L. in skin diseases. *T. terrestris* L. contains various bioactive metabolites, including steroidal saponins, flavonoids and alkaloids. These metabolites exhibit anti-inflammatory, antioxidant, antibacterial properties, and tyrosinase-regulating effects, making *T. terrestris* L. a promising candidate for treating multiple skin disorders. Studies have shown its potential efficacy against conditions such as atopic dermatitis, acne, and vitiligo. However, several limitations remain: its precise mechanisms of action in skin diseases are not yet fully elucidated, its standalone efficacy for complex skin diseases may be limited, and there is a lack of high-quality, large-scale clinical trials to conclusively verify its efficacy and safety. In conclusion, current evidence suggests *T. terrestris* L. has significant promise for the treatment of skin diseases. Future research should focus on conducting rigorous clinical trials, exploring combination therapies with conventional treatments, and deepening the investigation into its active components and mechanisms to expand its application in skin diseases.

## 1 Introduction

*Tribulus terrestris* L. (*T. terrestris* L.), a traditional medicinal plant, has been widely used in folk medicine for centuries. Belonging to the Zygophyllaceae family, a diverse and globally distributed group comprising 25 genera and approximately 250 species (Stefanescu et al., 2020). *T. terrestris* L. is a crawling herbal plant that grows in Mediterranean, subtropical, and desert climates around the world (William et al., 2009). *T. terrestris* L. is year-round flowering and every part of the plant has different therapeutic uses. Its fruit is considered a tonic diuretic and aphrodisiac, offering remedies for urinary diseases, impotence and heart disease. Its seeds are recommended for bleeding, kidney stones and gout (Shinwari and Khan, 2000). In addition to these applications, its roots have been demonstrated cardiotonic properties (Chhatre et al., 2014). These therapeutic benefits are attributed to *T. terrestris* L.'s diverse bioactive metabolites, such as saponins, flavonoids, and alkaloids, which contribute individually or synergistically to its pharmacological activities (Zhu et al., 2017).

The skin, as the largest organ of the human body, is central to physical health and well-being. Skin disorders not only affect the skin but may also involve hair, nails, and mucous membranes (Brown et al., 2024). Globally, skin diseases account for a substantial portion of healthcare needs. In developing nations, up to 70% of the population experiences skin-related issues. In developed countries, skin diseases account for 15%–30% of outpatient medical services (Ferreira et al., 2021). These disorders vary widely in severity, from benign ailments to rare and life-threatening diseases, often imposing substantial burdens on patients regarding both quality of life and financial costs (Petronic-Rosic, 2022). Although there are multiple therapeutic options, many skin diseases remain inadequately managed due to their complex pathogenesis. Therefore, the exploration of skin disease treatment is still ongoing, needed for attention.

As a well-known traditional Chinese medicine (TCM), *T. terrestris* L. is reputed for its ability to alleviate liver depression, promote blood circulation, dispel wind, and relieve itching (Gunarathne et al., 2023). With the development of modern research, its potential applications in skin diseases have begun to attract attention. However, comprehensive reviews focusing on *T. terrestris* L.'s role in managing skin diseases are limited. This paper aims to fill that gap by summarizing the therapeutic potential of *T. terrestris* L. for various skin conditions, offering insights into its role in the medical field of in skin diseases.

To ensure a comprehensive identification of all relevant literature, a systematic electronic search was conducted across the following major databases: PubMed, Web of Science, Scopus and Science Direct. The search strategy was built around three core conceptual blocks: 1) the plant source (*T. terrestris* L.), 2) its metabolites, and 3) skin-related pathologies and mechanisms. Keywords within each block were combined with the Boolean operator “OR”, and the three blocks were then intersected using “AND”. This approach ensured broad coverage of relevant studies. The search terms included: “*Tribulus terrestris* L.” AND (“skin diseases” OR “dermatology” OR “skin disorders”). The search was limited to studies published from database inception until December 2025. The primary objective was to identify studies that elucidate the phytochemistry of *T. terrestris* L. and its bioactive metabolites, with a specific focus on their mechanistic actions and therapeutic potential in the context of skin diseases. All retrieved records were imported into EndNote X20 software for duplicate removal. Any discrepancies between reviewers during the selection process were resolved through discussion or consultation with a third senior author.

## 2 Botanical characteristics of *Tribulus terrestris* L

### 2.1 Morphological features

**FIGURE 1:**
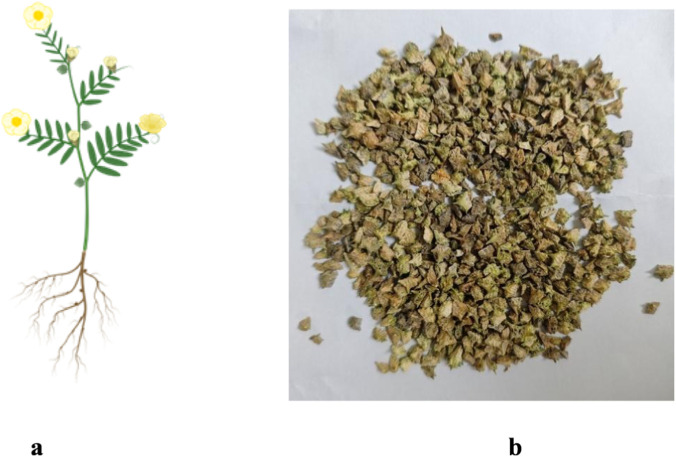
The morphology of *T. terrestris* L.: **(a)** Whole plant **(b)** Seed.

*T. terrestris* L. is a creeping herb with filamentous hairs that generally attains a height of 10–60 cm. The stem is either prostrate or ascending, typically exhibiting branching and capable of covering a relatively extensive area. Its length varies, generally adorned with fine hairs that impart a slightly rough texture. These hairs may play a role in protecting the plant from excessive water loss and also potentially from some herbivores. The leaves of *T. terrestris* L. are arranged oppositely and are typically pinnately compound, with each leaf comprising several pairs of leaflets. The leaflets are small and oblong-lanceolate in shape, featuring serrated margins. The upper surface of the leaves is often dark green, while the lower surface tends to be lighter in color, occasionally exhibiting fine hairs along the veins. The flowers of *T. terrestris* L. are small and yellow, occurring either solitarily or in small clusters at the leaf axils. Each flower comprises five petals and sepals (Stefanescu et al., 2020; Shahid et al., 2016). The fruit is a unique feature of this plant. The fruits are a faint greenish-yellow and possess spines. They are globose in shape, comprising five nearly glabrous, muriculate, wedge-shaped woody cocci, each featuring two pairs of hard, sharp spines, with one pair being longer than the other. Inside the capsule, there are several seeds, with horizontal spaces between them. The scent is lightly fragrant, and the taste is slightly spicy (Chhatre et al., 2014). The morphology of *T. terrestris* L. is detailed in Figure 1.

### 2.2 Geographic distribution and growth habits

*T. terrestris* L. is native to regions including Southern Europe, Southern Asia, Africa, New Zealand, and Northern Australia, thriving across tropical, subtropical, and temperate zones. This plant exhibits strong environmental adaptability, thriving in various soil types, including sandy, loamy, and even relatively poor-quality soils. While it prefers well-drained areas, it can also withstand certain levels of drought conditions. In addition, *T. terrestris* L. is a thermophilic plant to some extent. It can tolerate high temperatures and is well - adapted to the warm climate of tropical and subtropical regions. *T. terrestris* L. typically grows in open fields, along roadsides, and in disturbed habitats. The flowering period of *T. terrestris* L. extends from April to October, while the fruiting period occurs from May to October (Yabesh et al., 2014). In regions with adequate sunlight and optimal temperatures, it can exhibit vigorous growth, frequently establishing dense populations within local habitats (Zhu et al., 2017).

## 3 Metabolites of *Tribulus terrestris* L

The phytochemical analysis of *T. terrestris* L. identified the presence of saponins, flavonoids, alkaloids, glycosides, tannins, terpenoids, amide derivatives, amino acids, and proteins. Among the various types of metabolites, steroidal saponins and flavonoids are regarded as the most significant metabolites (Zhu et al., 2017).

### 3.1 Steroidal saponins

The steroidal saponins primarily consist of furostanol and spirostanol types (Wang Z. F. et al., 2016). It is believed that furostanol saponins serve as biogenetic precursors to their spiro analogs. Furostanol saponins have a characteristic five-membered furan ring attached to the steroid nucleus (Zhu et al., 2017). The structure of spirostanol saponins features a spiro configuration of rings. These saponins contain glycosidic bonds with various sugar moieties (e.g., glucose, rhamnose) attached to the aglycone part, which is the non-sugar metabolites typically consisting of a steroid or triterpene (Dabrowska-Balcerzak et al., 2021). The saponin composition and content of *T. terrestris* L. vary across different geographic regions. For example, protodioscin is a well - known furostanol saponin in this plant. Research indicates that prototribestin was exclusively detected in samples collected from Bulgaria, Turkey, Greece, Macedonia, Iran, and Serbia; conversely, no prototribestin was found in samples obtained from Vietnam and India (Dinchev et al., 2008). Saponins have several important biological functions. They have been demonstrated to possess anti-inflammatory effects through the activation of macrophages and other immune cells, thereby modulating the immune system (Passos et al., 2022). Some studies also suggest that saponins from *T. terrestris* L. may have potential benefits for sexual health, although the exact mechanisms related to this aspect are still under investigation (Kamenov et al., 2017; Salgado et al., 2017).

### 3.2 Flavonoids

Flavonoids are another important group of metabolites found in *T. terrestris* L. Studies indicated that the content of major flavonoids is about 1.5 times that of major saponins (Xu et al., 2010). The flavonoids present in *T. terrestris* L. predominantly consist of derivatives of quercetin, kaempferol, and isorhamnetin. The chemical structure features of quercetin lie in the hydroxyl group at the C-3 position, the double bond of C-2 = C-3, and the catechol structure on the B-ring, which are the keys to its powerful antioxidant activity (Xu et al., 2019). Studies have shown that quercetin is a classic activator of the nuclear factor E2-related factor 2 (Nrf2) pathway and can significantly enhance the activity of intracellular antioxidant enzymes (such as superoxide dismutase and catalase) (Cheng et al., 2024). Meanwhile, treatment with quercetin 5–15 mg/kgeffectively down-regulates the expression of key pro-inflammatory factors such as tumor necrosis factor -α (TNF-α) and interleukin-6 (IL-6) by inhibiting the nuclear factor κB (NF-κB) and mitogen-activated protein kinase (MAPK) signaling pathways in a dose-dependent manner (Wang et al., 2017). Kaempferol, as a methylation analogue of quercetin, lacks a hydroxyl group at the C-3′ position (Abdal Dayem et al., 2015). It has been demonstrated powerful anti-inflammatory and antiproliferative activities. Studies have confirmed that kaempferol ((25, 50, and 100 μM) can specifically inhibit the expression of cyclooxygenase-2 (COX-2) and inducible nitric oxide synthase (iNOS), thereby exerting anti-inflammatory effects in interleukin-1β(IL-1β)-stimulated rat OA chondrocytes (Zhuang et al., 2017). Flavonoids are well-known for their antioxidant activities. They can scavenge free radicals such as superoxide anions, hydroxyl radicals, and peroxyl radicals. This antioxidant property is crucial in protecting cells from oxidative damage, which is involved in many skin diseases and aging processes (Hu et al., 2017). Additionally, some flavonoids have shown potential antibacterial and analgesic activities, although more research is needed to confirm their role in disease prevention and treatment in the context of *T. terrestris* L. (Tian et al., 2019).

### 3.3 Alkaloids

Alkaloids represent a class of nitrogenous metabolites with significant pharmacological potential in *T. terrestris* L. The chemical characterization of *T. terrestris* L. utilizing UPLC-QTOF-MS in conjunction with ion mobility spectrometry revealed the presence of 20 distinct alkaloids within the fruit of *T. terrestris* L. (Meng et al., 2022). The beta-carboline indoleamines harmane and norharmane, recognized as two principal alkaloid metabolites of *T. terrestris* L., have demonstrated significant effects on the central nervous system (Bourke et al., 1992). They may have a calming and anti-anxiety effect to some extent by interacting with neurotransmitters in the brain, such as GABA receptors (Bourke et al., 1992). Additionally, the extract of *T. terrestris* L. alkaloids (70.2 and 140.4 μg/mL) can induce cancer cells to undergo apoptosis. Jurkat cells exposed to alkaloid extracts at sub-lethal concentrations exhibited DNA fragmentation, increased caspase-3 activity, and phosphatidylserine translocation (apoptosis indicator) when compared to control cells (Basaiyye et al., 2018). Some β-carboline alkaloids have demonstrated anti-proliferative effects on rapidly dividing cells. This property warrants investigation in hyperproliferative skin disorders like psoriasis, where keratinocyte turnover is excessively accelerated (Guo et al., 2024). However, more research is needed to fully understand their mechanisms of action and potential therapeutic applications.

### 3.4 Other metabolites

*T. terrestris* L. also contains other metabolites such as steroids, polysaccharides and fatty acids. Phytosterols, like β-sitosterol, possess a structure that closely resembles that of cholesterol, albeit with a distinct side-chain configuration (Li et al., 1998). The presence of these sterols may potentially influence the activity of hormones such as androgens and estrogens (Alexander et al., 2022; Hammoda et al., 2013). *T. terrestris* L. is also composed of polysaccharides, which are intricate carbohydrates consisting of multiple sugar units and may exhibit antioxidant properties (Van Quoc et al., 2024). In addition, this plant contains various organic acids, such as benzoic acid, vanillic acid, ferulic acid and succinic acid (Wang F. et al., 2016; Li, 2006). The therapeutic potential of *T. terrestris* L. in skin disorders cannot be attributed to a single compound but arises from the synergistic interplay of its multi-class phytochemicals. Further research is needed to fully understand the mechanisms of action and potential uses of these metabolites.

## 4 Mechanisms of potential protective effect in skin diseases

### 4.1 Anti-inflammatory

Inflammation is a key factor in the development of various skin diseases. In the pathological process, inflammatory cytokines can directly or indirectly damage skin tissues and cells, leading to congestion and exudative responses (Shirley et al., 2024). *T. terrestris* L. contains various bioactive metabolites, such as flavonoids, saponins, and alkaloids. These metabolites have been shown to exhibit comparable anti-inflammatory effects to the standard drug indomethacin in a carboplatin-induced edema model (Ahmad et al., 2012). Another study also reported the anti-inflammatory potential of *T. terrestris* L., where the root and fruit decoction of *T. terrestris* L. showed significant inhibition of carrageenan intoxicated swelling in rats, but its activity was slightly lower than that of the standard drug diclofenac sodium (Sudheendran et al., 2017).

*T. terrestris* L. modulates multiple inflammatory pathways, such as NF-κB and MAPK. For instance, its extract significantly inhibits the lipopolysaccharide (LPS)-induced transcription of inflammatory cytokines (TNF-α, IL-1β and IL-6) in RAW 264.7 cells—a well-established *in vitro* model for innate immune screening (Zhao et al., 2021). While findings from this cell line provide mechanistic insight, their direct relevance to human skin pathophysiology remains indirect. Evidence from *in vivo* models strengthens this translational rationale. For example, in a rat model of middle cerebral artery occlusion (MCAO), *T. terrestris* L. extract significantly reduced brain levels of TNF-α and IL-6 by modulating inflammation-related metabolic pathways, including glycerophospholipid and arachidonic acid metabolism (Zhang et al., 2023). At the molecular level, specific constituents contribute to its anti-inflammatory profile. N-trans-ρ-caffeoyl tyramine isolated from *T. terrestris* L. has been identified as a novel inflammatory agent that acts by suppressing the expression of inflammatory mediator cyclooxygenase-2 (COX-2) and prostaglandin E2 (PGE2) (Ko et al., 2015). Furthermore, the fruit extract of *T. terrestris* L. can reduce mast cell degranulation and T-cell activation in LPS-stimulated RAW 264.7 macrophages by inhibiting Orai-1 and TRPV3 channels, which can impact pruritus and inflammation in eczematous conditions (Kang et al., 2017). Collectively, the ability of *T. terrestris* L. extract to potently suppress macrophage activation through core signaling inflammatory pathways provides a compelling mechanistic premise for its potential application in treating macrophage-mediated cutaneous inflammation. Nevertheless, direct evidence in mammalian skin disease models remains necessary to confirm this therapeutic potential.

### 4.2 Antioxidant

Oxidative stress arises from an imbalance between prooxidants and antioxidants, ultimately leading to cellular damage (Liu et al., 2023). As a barrier organ, the skin is highly susceptible to oxidative stress, which is implicated in the pathogenesis of numerous skin diseases (Baek and Lee, 2016). The antioxidant properties of *T. terrestris* L. may help shield the skin from free-radical-mediated damage. *In vitro,* its methanol extract exhibits significant antioxidant activity in DPPH, FRAP and H_2_O_2_ assays (Abbas et al., 2022). However, such *in vitro* antioxidant capacity does not necessarily translate directly to cytoprotective or systemic effects *in vivo*.

Evidence from animal and clinical studies supports its antioxidant potential in living systems. In a streptozotocin (STZ) -induced diabetic rats model, an ethanol extract of *T. terrestris* L. reduced the levels of malondialdehyde (MDA) and glutathione (GSH) (Amin et al., 2006). Furthermore, a randomized controlled clinical trial involving 13 healthy men reported that 4-week supplementation with *T. terrestris* L. significantly attenuated oxidative stress responses (Ataei et al., 2023).

Further mechanistic insight comes from cellular models of oxidative injury. Furthermore, *T. terrestris* L. has been shown to alleviate oxidative stress-induced injury in the human retinal pigment epithelial (ARPE-19) cells via the PI3K/Akt-Nrf2 signaling pathway. After treatment with *T. terrestris* L. ethanol extract (100 and 200 μg/mL) for 24 h, H_2_O_2_-induced up-regulation of reactive oxygen species (ROS) activity and downregulation of superoxide dismutase (SOD) activity were significantly reversed (Yuan et al., 2020). The ARPE-19 cell line is a well-established *in vitro* model for studying oxidative damage, particularly in the context of age-related macular degeneration (Peterson et al., 2024). While this model is ophthalmology-specific, the mechanisms of oxidative stress it reveals are fundamental. Thus, findings from ARPE-19 cells may provide indirect mechanistic insights for future research into *T. terrestris* L. for managing oxidative stress-related dermatoses, such as those involving photodamage or inflammatory hyperpigmentation. Finally, various extracts of *T. terrestris* L.—including flavonoids, polyphenolic carboxylic acids, and saponins—have been extensively demonstrated to possess antioxidant properties (Stefanescu et al., 2020; Figueiredo et al., 2021).

### 4.3 Antibacterial activity

*T. terrestris* L. exhibits broad-spectrum antimicrobial activity, supported by both experimental and preliminary clinical evidence. An *in vitro* antibacterial assay demonstrated that its extract exhibited significant antibacterial activity against six pathogens, including *Actinomyces* viscosus, *Streptococcus* mutans, *Streptococcus* sanguinis, *Staphylococcus aureus*, and *Escherichia coli* (Soleimanpour et al., 2015). The antimicrobial mechanisms may involve the inhibition of key bacterial enzymes, such as DNA gyrase and topoisomerase, thereby disrupting DNA replication and transcription to suppress bacterial growth.

Furthermore, research indicates that metabolites from *T. terrestris* L., particularly saponins, exert antifungal effects by directly interacting with fungal cell membranes. This interaction disrupts membrane integrity, leading to the loss of intracellular components and ultimately cell death. For example, saponins isolated from *T. terrestris* have been shown to effectively destabilize the cell membrane of *Candida* albicans, contributing to its eradication (Zhang et al., 2006). Notably, a clinical trial suggests translational potential. In a trial involving 127 women aged 18–50 with bacterial vaginosis, a Persian herbal vaginal suppository containing Tribulus (Forzejeh) exhibited therapeutic effects comparable to metronidazole (Baery et al., 2018). This finding, while in a non-dermatological context, provides clinical support for its antimicrobial properties.

### 4.4 Tyrosinase-regulating effect

Melanin is the primary pigment responsible for skin pigmentation (Lai et al., 2018). Tyrosinase is the key enzyme in melanin biosynthesis, catalyzing the conversion of tyrosine to dopaquinone. Dopaquinone then undergoes a series of enzymatic and spontaneous reactions to ultimately form melanin (Jin et al., 2024). Dysregulation of tyrosinase activity disrupts melanin formation and distribution, contributing to pigmentary disorders such as vitiligo and melasma (Pisano et al., 2024). *T. terrestris* L. demonstrates a complex influence on the melanogenic pathway, with evidence pointing to context-dependent effects. Preclinical studies have reported inhibitory actions. In a rat model, oral administration of a methanol extract of *T. terrestris* L. combined with turmeric inhibited tyrosinase activity by up to 72% (Abbas et al., 2023). Similarly, in B16F0 mouse melanoma cells, an herbal extract containing *T. terrestris* L. significantly reduced melanin production, tyrosinase activity, and associated signaling pathways (Ho et al., 2021). These findings suggest potential for reducing excessive melanin synthesis.

Conversely, other data indicate melanogenic stimulation. One study found that the extract from *T. terrestris* L. increased tyrosinase activity and melanin production by upregulating the microphthalmia-associated transcription factor (MITF) (Cao et al., 2024). An *in vitro* study further reported that a decoction could enhance tyrosinase activity at a high concentration (100 mg/mL) but inhibit it at a lower concentration (10 mg/mL), a phenomenon often described as “bidirectional regulation” (Deng et al., 2002). However, the concept of a direct, concentration-dependent “bidirectional regulation” of the tyrosinase enzyme requires stringent validation. This claim necessitates confirmation through comprehensive dose-response studies using purified compounds and physiologically relevant skin models. The apparent contradictory effects are more parsimoniously explained by *T. terrestris* L. acting as a multi-target modulator of the melanogenic network. The net outcome on pigment production likely depends on the specific phytochemical composition of the extract, its concentration, and the cellular or pathological microenvironment. The potential pharmacological effects and mechanisms of *T. terrestris* L. are detailed in Table 1.

**TABLE 1:** The potential pharmacological effect and mechanism of *T. terrestris* L.

Compound/Extract	Model	Key treatment & controls	Pharmacological activity	Mechanism	Reference
*T. terrestris* L. extract	RAW 264.7 macrophages (LPS-induced, *in vitro*)	30 μg/mL, 24 h Ctrl: Untreated, LPS	Anti-inflammatory	↓ NF-κB and MAPK signaling pathways	Zhao et al. (2021)
Gross saponins of *T. terrestris* L. fruit (>60% saponins)	MCAO rat model (*in vivo*)	200 mg/kg/day, p.o., 2 weeks Ctrl: Sham, MCAO + vehicle	Anti-inflammatory	↓ TNF-α, IL-6, ↓ TLR4/MyD88/NF-κB pathway	Zhang et al. (2023)
*N-trans-p*-coumaroyltyramine (isolated from TT)	LPS-stimulated RAW 264.7 macrophages (*in vitro*)	5, 25, 50 μM, 24 h Ctrl: Untreated, LPS, Dexamethasone	Anti-inflammatory	↓ COX-2, PGE_2_, TNF-α, IL-6, IL-10, ↓ Oral-1/TRPV3 channels	Ko et al. (2015)
*T. terrestris* L. ethanol extract (70%)	STZ-induced diabetic rats (*in vivo*)	2 g/kg, 30 days Ctrl: Normal + saline, Diabetic + saline, Diabetic + glibenclamide	Antioxidant	↓ ALT, AST ↑ GSH	Amin et al. (2006)
*T. terrestris* L. ethanol extract	H_2_O_2_-induced ARPE-19 cells (*in vitro*)	100, 200 μg/mL, 24 h Ctrl: Untreated, H_2_O_2_, LY294002 (PI3K inhibitor)	Antioxidant	Activates PI3K/Akt-Nrf2 pathway, ↑ Nrf2, SOD, HO-1, NQO1, GCLM	Yuan et al. (2020)
Steroidal saponins from *T. terrestris* L.	*Candida* albicans SC5314 (*in vitro*) and vaginal infection rat model (*in vivo*)	*In vitro*: 4.16 μg/mL, 6 h *In vivo*: 30, 60 mg/kg, multiple days Ctrl: Untreated, Fluconazole	Antibacterial activity	Inhibits hyphal/biofilm formation, reduces infection rate *in vivo*	Zhang et al. (2006)
Tribuloside (flavonoid from *T.terrestris* L.)	Human epidermal melanocytes (*in vitro*)	5–20 μM, 24 h Ctrl: Untreated	Tyrosinase-regulating effect	↓ PDE, ↑ cAMP, MITF, tyrosinase expression	Cao et al. (2024)

## 5 Application and future perspectives in skin diseases

### 5.1 Atopic dermatitis

Atopic dermatitis (AD) is a chronic, relapsing inflammatory skin disease characterized by intense pruritus, eczematous lesions, and a disrupted skin barrier (Sroka-Tomaszewska and Trzeciak, 2021). Its pathogenesis involves a complex interplay of immune dysregulation, primarily a T-helper 2 (Th2) and T-helper 22 (Th22) skewed response, and epidermal barrier defects (Haddad et al., 2022).

While clinical evidence is currently lacking, the pharmacological profile of *T. terrestris* L. provides a strong mechanistic rationale for its potential utility in AD management. A study evaluated the therapeutic effect of *T. terrestris* L. fruit extract by inducing a murine model of AD with oxazolone. The results showed that the combination of 1% *T. terrestris* L. extract and 0.1% hydrocortisone (HC) could significantly alleviate skin erythema, edema, and epidermal hyperplasia, reduce transepidermal water loss (TEWL) and symptom scores (Kang et al., 2017). Mechanistically, *T. terrestris* L. inhibited Orai-1 channel activity to suppress T cell activation, activated TRPV3 channels to promote barrier repair, and dose-dependently stabilized mast cells by inhibiting β-hexosaminidase release without cytotoxicity (Kang et al., 2017). Furthermore, the combination of *T. terrestris* L. with low-dose HC exhibited superior anti-inflammatory effects compared with hydrocortisone monotherapy, while reducing potential hormone-related toxicity (Kang et al., 2017).

Collectively, this research demonstrates that *T. terrestris* L. exerts multi-targeted anti-AD effects via regulating calcium channels and mast cell function, providing experimental evidence for developing natural adjuvant therapies and safe hormone combination regimens for AD treatment. The therapeutic potential of *T. terrestris* L. in AD is strongly supported by its multi-target pharmacological profile, which aligns with several key pathological drivers of the disease. On one hand, the potent anti-inflammatory and immunomodulatory properties of *T. terrestris* L. directly address the immune dysregulation in AD. Key flavonoids such as quercetin and kaempferol have been shown to suppress the activation of NF-κB and MAPK signaling pathways, thereby reducing the production of critical AD-associated cytokines, including IL-6, IL-8, and thymic stromal lymphopoietin (Beken et al., 2020; Lee and Jeong, 2021). By dampening this Th2-axis inflammation, metabolites of *T. terrestris* L. could alleviate pruritus and reduce the severity of eczematous lesions. On the other hand, the antioxidant activity of *T. terrestris* L. metabolites, primarily mediated through the Nrf2 pathway, is highly relevant (Yuan et al., 2020). Oxidative stress is a significant contributor to skin barrier damage and the exacerbation of AD (Raimondo et al., 2023). The antioxidant capacity of *T. terrestris* L. metabolites could help mitigate oxidative stress, which exacerbates barrier dysfunction and inflammation in AD. In conclusion, while *T. terrestris* L. is a medicinal plant with a mechanistically well-founded profile for AD, its translation from traditional use to evidence-based dermatological therapy awaits rigorous, disease-specific investigation focused on topical delivery and comprehensive efficacy validation.

### 5.2 Acne vulgaris

Acne vulgaris is a multifactorial inflammatory disorder of the pilosebaceous unit, primarily driven by four key pathogenic factors: excess sebum production, hyperkeratinization of the follicular infundibulum, colonization by Cutibacterium acnes, and the resultant inflammatory cascade (Mias et al., 2023). Up to 85% of teenagers and young adults are affected by this skin disease (Haz and arika, 2021).

Research has indicated that *T. terrestris* L. may have potential in the treatment of mild to moderate acne vulgaris. An 8-week, double-blinded, randomized, controlled trial evaluated herbal extracts containing *T. terrestris* L. for mild-to-moderate acne. 60 patients were randomized 1:1 to herbal extract (n = 60) or placebo (n = 60) topical preparation. After 8 weeks, the herbal group significantly reduced the incidence of both non-inflammatory and inflammatory acne lesions compared to the vehicle (Yang et al., 2021). The expression of IL-1α, IL-8, and keratin 16 in the herbal extract group was significantly decreased (Yang et al., 2021). In addition, only 6 cases in the herbal group had mild adverse events, such as burning sensation, confirming its efficacy and safety (Yang et al., 2021). *T. terrestris* L. has ability to attenuate the expression of LPS-induced inflammatory cytokines, including IL-6 and tumor necrosis factor-α, which are critical mediators in the inflammatory pathway associated with acne (Lee et al., 2017). The anti-inflammatory properties make it a promising candidate for treating acne.

### 5.3 Psoriasis

Psoriasis is a chronic autoimmune skin disease characterized by red, scaly patches on the skin (Liu et al., 2024). The pathogenesis involves excessive proliferation of keratinocytes and infiltration of immune cells, mainly driven by the IL-23/Th17 axis, and there is a lack of safe long-term treatment methods (Krzysztofik et al., 2024).

*T. terrestris* L. exhibits therapeutic potential via its bioactive metabolites (e.g., terrestriside D, gross saponins), which target psoriasis-related pathological pathways. Terrestrosin D (TED), as the effective monomeric metabolite of *T. terrestris* L., has been shown to have a beneficial effect on psoriasis. In an imiquimod-induced psoriasis-like murine model, TED significantly reduced the psoriatic area and severity index (PASI) scores, as well as epidermal thickness and Ki-67 expression levels (Guo et al., 2022). Furthermore, TED diminished dendritic cell (DC) maturation, downregulated the expression of inflammatory factors, and improved both skin lesions and behavioral outcomes in psoriasis-like murine models by inhibiting the interaction between Substance P and DCs (Guo et al., 2022).

Additionally, in a mouse model of psoriasis induced by oxazolone, the ears of the mice showed swelling, significant epidermal hyperplasia, and infiltration of inflammatory cells consisting of monocytes, granulocytes, and macrophages. The application of extracts of *T. terrestris* L. can lead to significant improvements in skin inflammation symptoms in mice and exert an anti-psoriatic effect. This might be associated with the inhibition of TNF-α produced by macrophages and interferon-γ generated by Th1 cells (Rajesh et al., 2013).

Despite promising results, challenges persist, such as unclear pharmacokinetics of *T. terrestris* L. and limited large-scale randomized trials. However, its advantages of multi-metabolite synergy and ability to address inflammatory make it a compelling candidate.

### 5.4 Vitiligo

Vitiligo is a common acquired depigmentation skin disease characterized by selective loss of melanocytes, resulting in white patches with irregular borders (Bialczyk et al., 2023). Its pathogenesis is multifactorial, involving susceptibility, autoimmune destruction mediated by CD8^+^ cytotoxic T-cells, and the heightened melanocyte vulnerability to oxidative stress (Perez-Bootello et al., 2023). Conventional treatments, including topical corticosteroids, calcineurin inhibitors, and phototherapy, often yield variable outcomes and carry risks of adverse effects or disease recurrence (Cunningham and Rosmarin, 2023).

*T. terrestris* L. show promising multi-target activity in experimental models of vitiligo. Tribuloside, a natural flavonoid extracted from *T. terrestris* L., significantly promotes melanin synthesis in melanocytes, zebrafish, and human skin samples (Cao et al., 2024). Mechanistically, its inhibitory effect on phosphodiesterase (PDE) activity, which elevates intracellular cyclic adenosine monophosphate (cAMP) and activates protein kinase A (PKA) (Cao et al., 2024). The activated PKA pathway ultimately promotes melanogenesis, melanocyte dendrite formation, and melanin transport without significant *in vitro* cytotoxicity (Cao et al., 2024).

Additionally, the water-based extract obtained from *T. terrestris* L. upregulates α-melanocyte stimulating hormone (α-MSH) and melanocortin-1 receptor (MC-1R) expression, promoting melanogenesis in mouse hair-follicle melanocytes (Yang et al., 2006). Moreover, as the main active metabolite in *T. terrestris* L., Kaempferol protects human primary epidermal melanocyte 1 (HEM-1) from RSL3-induced ferroptosis by reducing ROS/iron levels and upregulating glutathione peroxidase 4 (GPX4) (Li et al., 2025).​ By enhancing cellular antioxidant defenses, *T. terrestris* L. extract could theoretically protect residual melanocytes from oxidative stress and stabilize the local epidermal environment, a foundational step for any repigmentation strategy.

Clinical-oriented evidence further supports its translational potential. Yubai Wan, a hospital-prepared formulation containing *T. terrestris* L. and honey, has been widely used vitiligo patients (Wang et al., 2025). Its metabolite, quercetin (10 mg/kg, 50 mg/kg and 100 mg/kg), alleviated hydroquinone -induced vitiligo-like depigmentation in guinea pigs and promote melanosome recovery in a dose-dependent manner (Wang et al., 2025). The mechanism is that quercetin activates the NRF2/GPX4 signaling pathway, thereby exerting an anti-ferroptosis effect on vitiligo (Wang et al., 2025). Collectively, these findings elucidate key molecular mechanisms and provide a pharmacological rationale for exploring *T. terrestris* L. -based interventions in vitiligo.

### 5.5 Skin cancer

Skin cancer represents one of the most prevalent forms of malignancies, encompassing melanoma and non-melanoma skin cancers like basal cell carcinoma and squamous cell carcinoma (Hasan et al., 2023). While surgical interventions are often curative for localized non-melanoma skin cancers, advanced or metastatic disease, particularly melanoma, requires systemic therapies that can be limited by toxicity and resistance (Hyeraci et al., 2023). The B portion of ultraviolet (UV) light has long been acknowledged as the primary risk factor for the development of skin cancer (Bagde et al., 2018).

**FIGURE 2:**
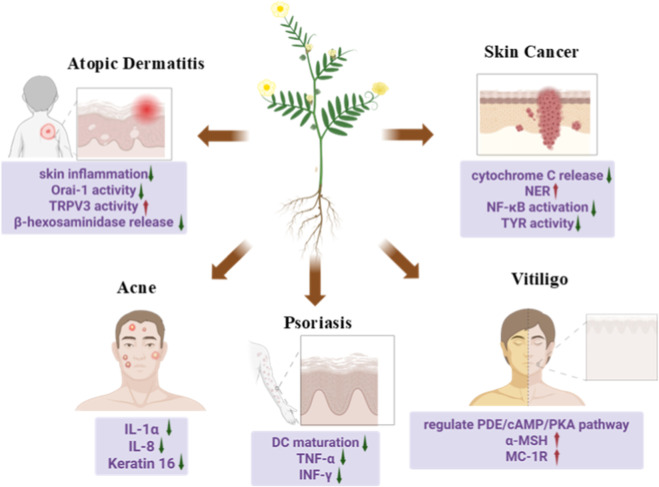
The application of *T. terrestris* L. in different skin diseases.

*T. terrestris* L. has shown potential in inhibiting UVB-induced skin carcinogenesis through multiple mechanisms. In normal human keratinocytes (NHEKs), the saponins derived from *T. terrestris* L. reduced the activation of caspases associated with the intrinsic apoptotic pathway, suppressed mitochondrial cytochrome C release, and decreased UVB-induced DNA fragmentation (Sisto et al., 2012). In squamous cell carcinomas (SCC), saponin prevented UVB-induced cell carcinogenesis by enhancing NER gene expression and blocking NF-κB activation (Sisto et al., 2012). Antioxidant-mediated chemopreventive effects have also been observed *in vivo*. Additionally, its antioxidant properties may prevent the formation of skin cancer induced by 7, 12-dimethylbenz(a)anthracene (DMBA) in mice. Following continuous oral administration of *T. terrestris* L. suspension, tumors incidence and tumor burden were significantly reduced, and the average latency period was notably prolonged. These effects were accompanied by a marked increase in hepatic reduced glutathione levels and a concurrent decrease in lipid peroxidation (Kumar et al., 2006). Furthermore, extracts of *T. terrestris* L. inhibit melanogenesis in B16F0 mouse melanoma cells by suppressing the activity of tyrosinase (Liu et al., 2022). Given the multifactorial nature of skin cancer, the greatest therapeutic potential of *T. terrestris* may lie in adjuvant or combination strategies. Its multi-target profile—simultaneously modulating oxidative stress, inflammation and apoptosis—makes it a compelling candidate to complement targeted therapies or immunotherapies, potentially improving efficacy and mitigating treatment-related adverse effects. The application of *T. terrestris* L. in various skin diseases is detailed in Figure 2.

## 6 Advantages and limitations of treating skin diseases

The potential benefits of *T. terrestris* L. in dermatology are supported by several key aspects. Firstly, its metabolites exhibit multi-target pharmacological activities, including anti-inflammatory, antioxidant, and tyrosinase-regulation effects. For instance, saponins alleviate inflammation, redness, and pruritus in conditions like eczema, while phenolic metabolites and flavonoids counteract oxidative damage (Li et al., 2024; Ma and Khachemoune, 2023). Secondly, as a plant-derived agent, *T. terrestris* L., is often perceived as a more natural alternative to synthetic drugs. Unlike long-term corticosteroid use, which may cause skin atrophy, telangiectasia, and other adverse effects, saponins extracted from *T. terrestris* L. have shown lower toxicity in normal human fibroblasts (Flori et al., 2023; Neychev et al., 2007). Finally, its longstanding use across various traditional medicine systems provides a historical basis for its therapeutic application and enhances its acceptability among patients seeking herbal remedies (Guo et al., 2020).

Notwithstanding these potential benefits, several important limitations in the current research warrant careful consideration. Currently, the most prominent constraint is the lack of high-quality, large-scale randomized controlled trials (RCTs) on the application of *T. terrestris* L. in the treatment of skin diseases. Consequently, the existing evidence level remains limited, and its clinical efficacy and safety lack robust prospective validation. Furthermore, the phytochemical profile of *T. terrestris* L. is subject to significant variability. The composition and concentration of key bioactive metabolites, such as alkaloids and saponins, can fluctuate considerably depending on the plant’s geographical origin, harvest time, and extraction methodology (Meng et al., 2022). This inherent variability may lead to inconsistent therapeutic outcomes.

Another critical limitation is its likely insufficient efficacy as a monotherapy for severe or complex dermatoses, such as cutaneous lupus erythematosus. The pathogenesis of such diseases involves intricate interactions between immune dysregulation, genetic factors, and environmental triggers, which are unlikely to be fully addressed by a single botanical extract. Therefore, future research should prioritize exploring rational combination strategies with conventional therapies (e.g., immunosuppressants, biologics) to assess potential synergistic effects and develop more effective integrated regimens. Finally, the translation of *T. terrestris* L. into clinical practice is hindered by the absence of standardized treatment protocols (e.g., dosages, concentrations, treatment duration) and incomplete safety profiles.

Despite its history of traditional use, *T. terrestris* L. is associated with documented toxicity and side effects that warrant careful consideration. Long-term safety data for topical formulations are currently lacking. Oral administration has been linked to gastrointestinal irritation, with case reports indicating potential hepatotoxicity and nephrotoxicity (Sun et al., 2022; Gama et al., 2014). Furthermore, its pharmacological activities suggest a potential risk of drug interactions, for example with antihypertensives, diuretics, or anticoagulants such as clopidogrel (Vatankulu et al., 2012; Al-Ali et al., 2003; Zhang et al., 2022). Therefore, a comprehensive safety assessment specific to dermatological formulations of *T. terrestris* L. is urgently needed.

## 7 Conclusions and prospects

*T. terrestris* L., as a traditional medicinal plant, shows considerable promise for dermatological therapy. Its efficacy is attributed to diverse pharmacological activities—including anti-inflammatory, antioxidant, antimicrobial, and tyrosinase-regulating effects—which underlie its therapeutic potential against a range of skin disorders. Bioactive metabolites such as glycosides, flavonoids, and alkaloids contribute to these benefits. Research demonstrated that *T. terrestris* L. may be effective in managing eczema, acne, psoriasis, vitiligo, and even skin cancer, supporting its potential integration into modern dermatological practice.

To advance the clinical translation and application of *T. terrestris* L. in dermatology, several research priorities should be addressed. First, conducting high-quality, rigorously designed controlled clinical trials are urgently needed to establish robust evidence for its efficacy and safety. Second, exploring combination strategies—integrating *T. terrestris* L. with other herbal agents or conventional therapies such as antihistamines and antibiotics—may enhance therapeutic outcomes and reduce recurrence rates. Third, further studies on its composition and pharmacological mechanisms are essential to identify its key active metabolites and support the development of standardized topical or oral formulations with consistent quality and demonstrated activity.

Looking ahead, advances in precision medicine could further refine its application. Genetic testing and biomarker analysis may help identify patient subgroups most likely to respond to *T. terrestris* L.—based therapies, paving the way for personalized treatment approaches that maximize clinical benefit and solidify its role in dermatological care.
